# Unintended consequences of the potential phase-out of gamma irradiation

**DOI:** 10.12688/f1000research.14090.1

**Published:** 2018-03-21

**Authors:** Jacquelyn W Chou, Michelle Skornicki, Joshua T Cohen

**Affiliations:** 1 Precision Health Economics, Los Angeles, CA, USA; 2 Tufts Medical Center, Boston , MA, USA

**Keywords:** Sterilization; Co-60; gamma irradiation

## Abstract

The radioisotope cobalt-60 (Co-60) is important for commercial, medical, and agricultural applications. Its widespread use has meant that Co-60 can be found in less secured facilities, leading to the fear that unauthorized persons could obtain and use it to produce a “dirty bomb”. This potential security concern has led to government calls for phasing-out Co-60 and other radiation sources, despite ongoing safety and security regulations for handling, transport and use of radioactive sealed sources.

This paper explores potential implications of phasing out radioisotopic technologies, including unintended safety and cost consequences for healthcare and food in the US and globally.

The use of Co-60 for healthcare and agricultural applications is well-documented. Co-60 is used to sterilize single-use medical devices, tissue allografts, and a range of consumer products. Co-60 is used in Gamma Knife treatment of brain tumors in over 70,000 patients annually. Co-60 is also used to preserve food and kill insects and pathogens that cause food-borne illness.

Co-60 is effective, reliable, and predictable. Limitations of alternative sterilization technologies include complex equipment, toxicities, incompatibilities with plastic, and physical hazards. Alternative ionizing radiation sources for wide-reaching applications, including e-beam and x-ray radiation, have advantages and drawbacks related to commercial scale capacity, penetrability, complexity and reliability.

Identifying acceptable alternatives would require time, costs and lengthy regulatory review. FDA testing requirements and other hurdles would delay replacement of existing technologies and slow medical innovation, even delaying access to life-saving therapies.  A phase-out would raise manufacturing costs, and reduce supply-chain efficiencies, potentially increasing consumer prices, and reducing supply.

These consequences are poorly understood and merit additional research. Given Co-60’s importance across medical and non-medical fields, restrictions on Co-60 warrant careful consideration and evaluation before adoption.

## Introduction

Naturally occurring cobalt is a stable element. One of its synthetic isotopes, Cobalt-60 (Co-60), has an extra neutron in its nucleus that makes it unstable. As it breaks down, Co-60 emits high energy, “ionizing” radiation that can break molecular bonds. Co-60 plays an important role in a wide variety of commercial, medical, agricultural and research applications because it is a “radioisotope” and hence generates steady, predictable ionizing radiation.

One of the most useful applications of radioisotopes in general and Co-60 in particular is sterilization. The list of single-use medical devices sterilized using Co-60 is lengthy, including surgical instruments, gloves, gowns, dressings, masks, catheters, laparoscopic equipment, implants, probes, and other objects that enter sterile tissue or the vascular system. Another major healthcare use of Co-60 is the sterilization of tissue allografts, including bone, skin, amniotic membrane and soft tissues used to treat severe burns, non-healing ulcers, and to facilitate organ transplants ^1, 2^. ( https://www.aatb.org/?q=about-us) Co-60 is also used to sterilize consumer products such as bottle teats for premature babies, medical bandages and a variety of personal health and hygiene products, and raw materials for cosmetics ^3, 4^. ( https://www.nei.org/Knowledge-Center/Other-Nuclear-Energy-Applications/Consumer-Products)

A second critical application of Co-60 is cancer treatment. Gamma Knife technology, developed in 1968, uses gamma radiation to target small brain tumors. By precisely targeting high doses of ionizing radiation generated by Co-60, Gamma Knife therapy can treat small brain tumors while mitigating damage to surrounding normal, healthy tissue. Gamma Knife therapy is used in over 70,000 patients annually ^5, 6^. ( http://gammaknife.com/what-is-gamma-knife/)

Co-60 has also been used since the 1920s to preserve food. ( https://www.fda.gov/food/ingredientspackaginglabeling/irradiatedfoodpackaging/ucm081050.htm.) By killing microorganisms, insects, and pathogens that can cause food-borne illness, such as salmonella and *escherichia coli (E. coli),* ionizing radiation extends food shelf life and improves food safety ^7, 8^. ( https://www.fda.gov/food/ingredientspackaginglabeling/irradiatedfoodpackaging/ucm081050.htm)

Because they produce radiation, radioisotopes have raised security concerns. The fear is that loss of radioisotopes – due to accident, oversight, or sabotage – could result in their acquisition by unauthorized persons or terrorists who could then produce a radiological dispersal device (“dirty bomb”) ^9^. Motivated by these concerns, some members of Congress proposed legislation in the 2015 Appropriations Bill that would have phased out the use of radioisotopes in the United States, including Co-60. ( http://www.nrc.gov/reading-rm/doc-collections/cfr/part020/) Though that legislation did not pass, the suggestion that use of Co-60 and other radioisotopes should be phased-out has persisted. In 2015, the Committee on Homeland and National Security created the Interagency Working Group on Alternatives to High-Activity Radioactive Sources (GARS) to develop best practices to transition to non-radioisotopic technologies.

This paper explores the merits and potential downside of shifting away from radioisotopic technologies. We describe unintended safety and cost consequences of a Co-60 phase-out for healthcare and food applications.

### Rationale for phase-out of Co-60

The widespread commercial use of Co-60 means it is often housed in less well-guarded facilities, such as hospitals, that are unlike heavily guarded nuclear facilities ^9^. Experience in other countries has demonstrated the potential for mishaps. For example, in 2013, thieves in Mexico stole a truck transporting Co-60. The thieves ultimately abandoned the Co-60, as the material itself did not appear to be the target, and the authorities recovered it ^10^. Also in 2013, online fashion retailer Asos recalled a batch of metal-studded belts contaminated with Co-60 likely introduced in scrap metal from India or another Asian country ^11^. In both of these cases, it was incidental exposure to Co-60 that posed the greatest public health risk, rather than the potential for a terrorist act. To date, these types of Co-60 mishaps have not been reported in the United States, perhaps because of this country’s stricter regulations. There have been no deaths from exposure to radiation or any history of contaminated groundwater at irradiators in the US. The US Nuclear Regulatory Commission (NRC) has reviewed cases of radiation incidents and has developed strict requirements designed to reduce future risk of incidents (10 CFR (Code of Federal Regulations) Part 36, implemented in 1993.) ( https://www.nrc.gov/reading-rm/doc-collections/fact-sheets/commercial-irradiators.html)

Ongoing regulatory changes and additional safety and security requirements have been implemented over the years to further increase and assure maintenance of safe and secure handling, transport and use of high activity radioactive sealed sources. The US NRC promulgated 10 CFR Part 37 in March 2013 with adoption required by US NRC licensees by March 2014 and Agreement State licensees by March 2016. ( https://www.nrc.gov/reading-rm/doc-collections/cfr/part037/) Part 37, “Physical Protection of Category 1 and Category 2 Quantities of Radioactive Material”, provides specific detail of control in the following areas:

i) Background investigation and access control program of people and facilities (criminal history checks and related other elements of personnel involved in handling, transport, and use of this material; access authorization to areas where such material is housed; personnel access authorization and control where material is used; and protection of information)
ii) Physical protection requirements during use (comprehensive security program for facilities and people; security zones; monitoring/detection/assessment; maintenance, testing; security program review; reporting of events)
iii) Physical protection in transit, including preplanning and coordination of transit; physical protection during transit; and personnel/vehicle controls
iv) Record keeping, and
v) Enforcement

### Desirable properties of Co-60

Co-60 has three main advantages. First, the gamma radiation it produces is versatile. Gamma radiation can deeply penetrate a wide range of low- and high-density materials ^1, 12, 13^. Deep penetration is important for sterilizing medical supplies and to reach pathogens deep within the food matrix ^14^. Because gamma radiation does not require high temperature, it can be used on temperature-sensitive items, products can be irradiated in bulk, and sterilization can take place after final packaging ^15, 16^.

Second, Co-60 is reliable. It has a simple and predictable decay pattern and a relatively long half-life (5.27 years). Treatment with Co-60 is precise and reproducible; ^15, 17– 19^ ( http://www.world-nuclear.org/information-library/non-power-nuclear-applications/radioisotopes-research/radioisotopes-in-medicine.aspx, http://www.world-nuclear.org/information-library/non-power-nuclear-applications/overview/the-many-uses-of-nuclear-technology.aspx) therefore, instrument calibration standards rely on Co-60 as a “gold standard” yardstick ^20^. Co-60 gamma irradiators are simple to use and control ^1, 3, 12^, and because the Co-60 itself generates the radiation, it is energy-efficient ^15^. These properties minimize operational maintenance requirements ^15^.

Finally, Co-60 has favorable physical characteristics that make it ill-suited to manipulation that could pose security risks. It cannot start a fission chain reaction, it is non-flammable, and it cannot poison a water supply because it is insoluble. Moreover, because Co-60 is not readily dispersible, it does not emit neutrons or leave residues, or cause other surrounding materials to become radioactive ^15, 16^.

### Limitations of Co-60 alternatives

Alternative sterilization technologies include chemical treatment, non-ionizing radiation, and other ionizing radiation sources ( Table 1). Chemical treatments pose a series of challenges. ^1, 21^. Ethylene oxide (EO) requires complex equipment, is toxic, and flammable, and poses an explosive hazard. EO can be inconsistent because its use depends on multiple variables—including temperature, time, pressure, vacuum, and concentration—to address differences in the target material’s physical characteristics (e.g., density and porosity), packaging, and humidity ^3, 12^. Peracetic acid-ethanol, although rapid ^22^ and compatible with a wide variety of materials, significantly reduces biomechanical strength, decreases remodeling activity in ligament grafts ^1^ and does not reduce infection risk ^22^. Heat treatment damages many materials. For example, steam autoclaving damages plastics which comprise many single-use items. Although evidence is limited and it can only treat heat-resistant materials ^22^, microwave treatment has been shown to effectively sterilize some bone allografts ^1^.

**Table 1.** Comparison of Cobalt-60 Sterilization Alternatives.

Type of Sterilization	Advantages	Challenges
Gamma radiation	• Can be used to sterilize health care products on a commercial scale ^1^ • Simplicity and reliability of irradiation equipment, the radiation source and ability to match source strength to production throughput ( http://www. iaea.org/inis/collection/NCLCollectionStore/_ Public/07/220/7220308.pdf#page=280) • Scalability for different throughput • Reactor-produced from metal ^59^Co, and therefore has a finite production cost ( http://www. iaea.org/inis/collection/NCLCollectionStore/_ Public/07/220/7220308.pdf#page=280)	• Not suitable for small scale ^22^ • Requires requalification of irradiator operation after source replenishments ^23^ • Some deleterious effects on patient-care equipment associated with gamma radiation include induced oxidation in polyethylene and delamination and cracking in polyethylene knee bearings ^22^
Electron beams (E-beam)	• Can be used to sterilize health care products on a commercial scale ^1^ • Near instantaneous dose delivery • Scalability for different throughput • Capability to integrate in an on-line process ^3^ • Short processing time ^23^	• Higher costs for accelerator investment and operations than gamma – not suitable for small scale ^22^ • Complex irradiation equipment design and higher maintenance costs / downtime than Co-60 • Low penetrability (bulk densities up to 0.25 g/cm3) ^23^ • Dose distribution through the irradiated product is less uniform than with gamma radiation ^1^
X-rays	• Comparable penetration to gamma rays ^3, 23^ • Recent developments in high current e-beam accelerators for X-rays ^3^ make it more practical	• Limited use, uncertain operating and usage cost estimates ^3^ • Higher costs for accelerator investment and operations than e-beam and gamma – not suitable for small scale ^22^ • Complex irradiation equipment design and potentially higher initial capital costs than gamma and higher maintenance costs / downtime than E-beam and Co-60 ^23^ • Accelerator source used for x-ray is less reliable than Co-60 for cargo container contraband and security screening applications ( http://www. iaea.org/inis/collection/NCLCollectionStore/_ Public/29/057/29057259.pdf)
Ethylene oxide	• Widest range of material compatibility except for moisture and temperature-sensitive materials (>30 degrees C and/or <30% RH) ^23^	• Hazardous (toxicity issues, explosive) ^23^ • Long processing time ^23^ • Many variables to control (temperature, time, pressure, vacuum, gas concentration, packaging and humidity) ^3, 23^; • Time-consuming for routine use between patients ^22^ • Package and all parts of product to be sterilized must be gas permeable, irrespective of density ^23^
Steam	• Preferred for aqueous preparations only ^3^; • Economical and short processing time; • Nontoxic and safe for the environment ^12^	• Strict temperature and moisture controls; • Many variables to control (temperature, time, pressure, vacuum, packaging and humidity) ^3^; • Cannot be used for heat-sensitive materials ^22^
Peracetic acid- ethanol	• Established sterilization of bone, dermis and amniotic membrane transplants with no evidence of impaired transplant properties ^1^ • Rapid sterilization time ^22^ • Less damaging process to delicate materials than steam; • Compatible with a wide variety of materials-plastics, rubber, and heat-sensitive items; • Single-use process, there is no possibility of contamination ^12^ • Faster cycle times than EO ^22^	• Has caused significantly reduced biomechanical strength and decreased remodeling activity in anterior cruciate ligament reconstruction tendon grafts ^1^ • Lack of evidence on reduction in infection risk and link to improved patient care ^22^
Thermodisinfection	• Found to preserve tensile strength necessary for clinical purposes ^1, 27^	• Small-scale
Microwave	• Effective for sterilization of bone allografts processed from femoral heads contaminated with Gram-positive and Gram-negative bacteria ^1^	• Lack of evidence on efficacy ^1^ • Can only be used with items that do not melt ^22^

Alternative ionizing radiation sources have both advantages and drawbacks. Like gamma radiation, e-beam can sterilize health care products on a commercial scale ^1^ and is currently used in many large facilities. E-beam delivers radiation rapidly and can be scaled ^3^. However, because the radiation is machine-generated, rather than a material by-product (as is the case with Co-60-generated gamma radiation), the equipment is complex and costly to install and operate. Nor is e-beam radiation as predictable or uniform as Co-60 gamma radiation ^1^. Finally, e-beam radiation does not penetrate materials as well as gamma radiation. X-ray radiation achieves penetration comparable to that achieved by gamma rays ^3^. However, similar to equipment used to generate e-beam, x-ray generating equipment is complex, expensive and less reliable than Co-60, with very few such sterilization units operating globally ^3, 13, 17, 23^. ( http://www.world-nuclear.org/information-library/non-power-nuclear-applications/overview/the-many-uses-of-nuclear-technology.aspx, http://www.iaea.org/inis/collection/NCLCollectionStore/_Public/29/057/29057259.pdf)

## Impacts – Medical – United States

Because ionizing radiation has desirable properties and radioisotopes reliably generate this type of radiation, limiting the use of radioisotopes could have implications for medical care, including single-use medical supplies, allografts, and therapeutic technologies. These impacts extend beyond the US population since the US supplies a significant amount of sterile medical devices globally.

### Sterilization of single-use medical supplies

Current estimates indicate that the sterilization industry is divided between EO (50%), gamma (40.5%), e-beam (4.5%) and other (including x-ray) (5%), and there are 200 healthcare facilities worldwide with commercial gamma sterilization capability ^23^. Co-60 is the most widely used form of radiation for sterilizing single-use medical products ^16^, as heat sterilization is often damaging, and alternative methods might not achieve sufficient penetration ^17, 23^. ( http://www.world-nuclear.org/information-library/non-power-nuclear-applications/overview/the-many-uses-of-nuclear-technology.aspx) Single-use medical products were used in over 52 million surgical procedures in the US in 2011, with 45% of single-use products sterilized using Co-60 ^23, 24^.

Curtailing radioisotope sterilization would make availability of some single-use products uncertain, hence potentially jeopardizing millions of surgical procedures yearly. At the very least, products for which the effectiveness of alternative sterilization technologies has not been established would have to undergo significant and both time and cost intensive testing ^25, 26^. In the case of products for which adequate sterilization proved infeasible, single-use medical products could become unavailable going forward. Predicting the impact of this disruption on health is difficult. We do not know how many technologies would be affected, for how long, or the adequacy of substitute or redesigned products. However, with the possibility that millions of surgeries could be affected, it is clear that curtailing use of radioisotope materials for single-use medical supply sterilization could be highly disruptive.

### Tissue allograft sterilization

More than 2 million allografts each year support more than 1 million annual tissue transplants ^2^. Used in reconstructive surgery for musculoskeletal injuries, allografts avoid the major complications associated with use of autogenic materials ^1^. In addition, allograft skin and amniotic membrane have unique properties that make them irreplaceable and indispensable in the treatment of serious burn injuries ^1^. The risk of transmitting infectious disease from donor to recipient necessitates sterilization.

Sterilization alternatives for tissue allografts (ethylene oxide, peracetic acid-ethanol, thermo-disinfection), microwave, electron beam) lack the same demonstrated effectiveness as gamma radiation ^1, 21^. Studies have identified insufficient penetration as a key limitation of alternative sterilization technologies ^1^. Although some evidence indicates that microwave sterilization is effective for bone allografts ^28^, evidence supporting microwave sterilization in general, and its use for other tissues is limited compared to the evidence for gamma radiation.

Even if more extensive evidence were available, a phase-out of radioisotope sterilization could trigger FDA regulatory testing requirements ^21^ and delay replacement of existing sterilization technologies, potentially affecting the supply of tissue allografts.

### Gamma knife surgery

Gamma Knife is a stereotactic radiosurgery technique that relies on Co-60 ^6^. Approximately 70,000 Gamma Knife surgeries take place worldwide each year, with nearly 1 million surgeries having been conducted from 1991–2013. ( https://gammaknife.com/downloads/Facts%20in%20short_1028438.01.pdf) While alternatives exist, the Gamma Knife technique, described in Table 2, is the most established, well-researched and validated form of radiosurgery. ( http://nyulangone.org/locations/center-for-advanced-radiosurgery/gamma-knife-radiosurgery)

**Table 2.** Stereotactic Radiosurgery Alternatives ( http://www.irsa.org/radiosurgery.html).

	Cobalt-60	Particle beam	Linear accelerator base
Treatment Area	Ideal for small brain tumors (less than 3.5cm) and functional disorders of the brain	Brain tumors and body cancers	Large brain tumors (over 3.5) cm, body cancers, head, and neck
Length of Treatment	One-day treatment	Multiple day treatments	Several sessions
Amount of Supportive Evidence	Available for over 40 years, substantial amount of published research supporting efficacy and usage	Little supportive research due to cost of a facility	Lack of peer-reviewed research about diagnosis and treatment
Additional Benefits	Better targeting Less damage to healthy tissue Fewer complications		More flexibility over larger areas and spreading treatments over several days
Common Brand Names	Gamma Knife	--	Novalis Tx, CyberKnife, TomoTherapy

Gamma Knife is specifically indicated for brain surgeries. Radiosurgery using Co-60 is non-invasive and accurate to 0.15mm. Because it can target small areas, Gamma Knife can be used more extensively than competing technologies that deliver larger tissue doses of radiation because they cannot be as finely focused ^29, 30^. The more precise targeting achieved by Gamma Knife also causes less damage to healthy tissue, speeding recovery and minimizing side effects ^31^. In a prospective cohort study in which physicians assigned and treated patients with either gamma knife radiosurgery or whole brain radiotherapy (follow-up of 1200 days, or 3.3 years), the mortality rate was lower for Gamma Knife patients (74.4% vs. 97.1%), and the median survival time greater (9.5 months for Gamma Knife versus 8.3 months for whole brain radiotherapy patients) ^32^. With approximately 70,000 gamma knife surgeries each year ^6^ and 1.2 added months (9.5-8.3) gained per surgery, eliminating Gamma Knife could potentially cost 7,000 life-years annually. Curtailing Co-60, which the Gamma Knife depends on, would eliminate the only demonstrated treatment for certain tumor types.

### Slowed innovation

A ban on radioisotope sterilization technologies could also slow medical innovation. Modifications to sterilization modalities could involve costly redesign and require additional validation for the sterilization process, the sterilization product itself, and its packaging ^23^. First, altering the sterilization method constitutes a major change to new drug applications (NDA) and abbreviated new drug applications (ANDA), requiring FDA approval prior to distribution of drug products ^26^. The added effort could divert resources away from development of new technologies. Alternative sterilization methods may require new 510(k) or premarket applications (PMA). For 510(k) applications, the FDA determines whether the device is at least as safe and effective as a legally marketed device (“substantial equivalence”) ^5, 8^. Although the process is supposed to take no more than 90 days, one sterilization company noted that the full FDA clearance process lasts 9 months. ( http://www.revoxsterilization.com/sites/default/files/Revox_OsteoArticle.pdf) The FDA believes that novel sterilization technologies “carry a substantial risk of inadequate sterility assurance if not conducted properly”. ( https://www.fda.gov/downloads/MedicalDevices/.../ucm109897.pdf) Therefore, recent guidance indicates that in the context of a novel sterilization process, FDA intends to inspect manufacturing facilities before clearing a 510(k). ( https://www.fda.gov/downloads/MedicalDevices/.../ucm109897.pdf) PMA applications or supplements require up to 180 days of review time, depending on the product’s regulatory classification, design, and other required changes ^5^.

Second, because these reviews will divert FDA’s resources, FDA’s approval of other new technologies will likely slow. The FDA already has a backlog of products awaiting approval. Further slowing the overburdened approval system could delay patient access to life-saving therapies ^33^. ( https://www.cato.org/publications/commentary/fda-can-be-dangerous-health)

Third, a Co-60 phase-out will raise manufacturing costs. It costs on average $31 million to bring a low-to-moderate 510(k) product from concept to market, with approximately three-quarters of the cost related to FDA-dependent or related activities. Costs for PMAs are $94 million, with $75 million related to the FDA process ^34^. By raising costs, a Co-60 phase-out could disincentivize future innovation.

Finally, Co-60 sterilization facilities are often located near medical device manufacturers or distribution hubs. Phasing out of these facilities would reduce the attendant supply chain efficiencies, potentially increasing consumer prices, delaying product availability, and reducing supply.

## Impacts – Non-Medical – United States

Because of the reliability and predictability of Co-60 for sterilization, its use extends beyond medical products. A phase-out has implications for both the food supply and the multitude of consumer goods that use Co-60 for sterilization.

### Food products

The US food supply relies heavily on gamma radiation. Co-60 is used for food preservation, shelf-life extension, and reduction of food-borne illness for domestic and international food products, including microbial disinfection of spices. ( http://www.fda.gov/NewsEvents/Newsroom/PressAnnouncements/ucm279485.htm) Most spices sold in the US are grown overseas in developing countries, where pollution and water issues can contaminate food shipped to this country. ( http://www.washingtonpost.com/wp-dyn/content/article/2010/03/13/AR2010031301111.html) Irradiation is also used as a quarantine treatment for fresh horticultural commodities, and as a substitute for fumigants in Asian countries and the US ^7^. ( https://uw-food-irradiation.engr.wisc.edu/Process.html)

Phasing-out Co-60 will likely affect US and global food supply chains as alternatives are established. Alternatives to Co-60 have characteristics that limit their ability to treat all food products requiring irradiation. Because gamma irradiation can completely penetrate a product, it can deactivate both surface pathogens and those found within the food matrix (see Table 3) ^14^. With almost a quarter of the world’s food irradiation units located in the US ^35^, switching to alternatives to Co-60 irradiation may cause some disruption to the global food supply system. There is potential for impacts to food prices, supply, and access, depending on how quickly the global food irradiation system would be able to respond to a US phase-out.

**Table 3.** Sterilization Alternatives for Food Products.

Type of Sterilization	Advantages	Challenges
Gamma rays	Penetrate fully to reach surface pathogens and those found within the food matrix ^14^ ; insignificant rise in temperature; high penetrating power; simple process to control ^3^	
Electron beam (E-beam)	Sterilization effectiveness comparable to gamma rays ^3^	Limited depth; Limited by the penetration of electrons (which is proportional to the accelerator) ^3^
X-ray	Comparable penetration to gamma rays ^3^	Limited use, uncertain cost estimates (based on 2008 IAEA review) ^3^

### Decommissioning costs

Like food products, consumer goods prices will likely increase in response to a Co-60 phase-out, as decommissioning current Co-60 facilities will be costly. In February 1999, a decommissioning project commenced for a gamma irradiation facility at the Brookhaven National Laboratory (BNL), located on Long Island, New York ^36^. The decommissioning process was involved, taking over a year to conduct three main phases: 1) preparation of the facility; 2) packaging and shipment of irradiation sources for disposal; 3) disposal/discharge of pool water (used for cooling) and dismantling. Ultimately, all 24,000 curies of cobalt-60 were removed.

Building replacement facilities is also resource-intensive, with food irradiation facilities estimated to cost between $3-5 million, ( https://uw-food-irradiation.engr.wisc.edu/Process.html) and the equipment alone required for an electron accelerator for medical device sterilization estimated to cost between $1 and $2 million ^37^. There is little publicly available information on the full spectrum of cost for Co-60 facilities, including facilities large enough for medical sterilization, but conservatively, they likely cost several million dollars. Consumer products, medical products, and food products that involve Co-60 for sterilization would all be affected by the cost of decommissioning and construction of new facilities for replacement sterilization.

## Worldwide impacts

Restrictions on Co-60 in the US may shift its use to other countries where weaker regulations may have additional economic and security consequences ^38^. At a recent IAEA conference on Safety of Radiation Sources and the Security of Radioactive Materials, held in 2000, countries submitted reports describing their use of radiation ^38^. Though the regulatory and enforcement status of these countries has likely improved since 2000, the conference proceedings identified several countries with serious regulatory limitations. Angola had begun to use radiation sources, including Co-60, but lacked appropriate infrastructure to control their sources, relying on technical assistance from IAEA and other Member States. Bangladesh was facing financial and administrative hurdles to train and motivate personnel, and to create necessary infrastructure and facilities to achieve safety standards compatible with IAEA International safety standards. To the extent that a national-security motivated phase-out of Co-60 applications in the US shifts Co-60 use to other countries, such changes could aggravate security risks.

A Co-60 phase-out in the US may also increase the cost (and reduce quality) of medical devices and therapies elsewhere. Fifty-percent of the world’s sterile single use medical devices come from the US. ( http://documentslide.com/documents/1-a-profile-of-the-radiation-source-sector-committee-on-radiation-source-use.html) Depending on the implementation details of a phase-out and transition, there could be an initial decrease in supply of single-use medical devices from the US, as well as a price increase. This outcome could affect the safety and efficacy of healthcare in other countries, particularly those with more limited resources.

## Discussion

The effectiveness, reliability, and predictability of Co-60 have made it a primary source of gamma irradiation for a wide variety of medical and non-medical applications in the US. Its widespread use, though a strength, has also meant that Co-60 can be found in less secured facilities. This potential security concern has led to calls to phase out Co-60 and other radiation sources.

These concerns should be considered in the context of trade-offs that Co-60 restrictions in the US would impose. These trade-offs include unintended consequences for both medical care and consumer access to products in the US and worldwide. The use of Co-60 for Gamma Knife surgery, sterilization of tissue allografts, and sterilization of single-use medical devices is highly effective and well-documented. Although there are alternative sterilization technologies, they all have limitations. Similarly, Co-60 is used across a range of food products, helping to maintain the quality and safety of food supplies in the US and abroad. Even if acceptable alternative technologies are identified, identifying those alternatives would take time and necessitate costly and lengthy regulatory review.

Just in the US, a phase-out of Co-60 would impose direct monetary costs, time costs, and limitations to access. However, a US phase-out could also shift gamma irradiation processing offshore, particularly for food processing. The establishment of additional Co-60 facilities in countries that may lack rigorous safety and security regulations on par with the US could exacerbate security concerns.

These consequences are not well understood and merit additional research. First, a systematic risk assessment and a cost-benefit analysis of a Co-60 phase-out should be undertaken. The potential trade-offs described in this paper should be quantified and weighed against risk and potential cost of security failure scenarios. An evaluation of efficacy, implementation timeline, and cost for Co-60 alternatives should be included.

Second, a comprehensive assessment of the risk of all radioactive isotopes should be undertaken, along with an evaluation of additional regulatory steps that could shore up security without a complete ban on use of these radiation sources. US regulations for transport, storage, and security already provide a measure of safety. However, given the recent calls for a complete phase-out, it appears more can be done to further improve security.

Lastly, how a phase-out of Co-60 in the US might influence the shift of Co-60 facilities to locations abroad, and how the spread of Co-60 use might influence the threat posed should be carefully evaluated. A follow-up meeting to the year 2000 Safety of Radiation Sources and the Security of Radioactive Materials conference could help to assess the regulatory progress made in each country.

National security concerns are always important, but they can be difficult to assess, particularly when it comes to preventing an event that has yet to occur. However, it is possible to assess the current use of Co-60 in the US and the impact of a potential phase-out. Given its importance across medical and non-medical fields, restrictions on Co-60 merit careful consideration and evaluation before their adoption.

## References

[1] Singh R Singh D Singh A: Radiation sterilization of tissue allografts: A review. *World J Radiol.* 2016;8(4):355–69. 10.4329/wjr.v8.i4.355 27158422 PMC4840193

[2] American Association of Tissue Banks: About Us.2016; [cited 2016 October 28]. Reference Source

[3] Trends in Radiation Sterilization of Health Care Products. International Atomic Energy Agency.2008 Reference Source

[4] Nuclear Energy Institute: Consumer Products. Reference Source

[5] US Food and Drug Administration: Premarket Notification PMA.2017.

[6] Elekta: What is Gama Knife?2016; [cited 2016 October 27]. Reference Source

[7] Morehouse KM Komolprasert V: Irradiation of Food and Packaging: An Overview.2004 Reference Source

[8] US Food and Drug Administration: Premarket Notification 510(k).2017 Reference Source

[9] Capala J Goetsch SJ Orton CG: Point/Counterpoint. Medical use of all high activity sources should be eliminated for security concerns. *Med Phys.* 2015;42(12):6773–5. 10.1118/1.4934823 26632034 PMC5148163

[10] Martinez G Partlow J: Stolen cobalt-60 found in Mexico; thieves may be doomed. *The Washington Post.* 2013 Reference Source

[11] Neville S: Asos pulls belts in radioactive scare. In *The Guardian* 2013.

[12] Silindir M Ozer Y: Sterilization Methods and the Comparison of E-Beam Sterilization with Gamma Radiation Sterilization. *FABAD J Pharm Sci.* 2009;34:43–53. Reference Source

[13] Interagency Working Group on Alternatives to High Activity Radioactive Sources (GARS): Transitioning from high-activity radioactive sources to non-radioisotopic (alternative) technologies. A best practices guide for federal agencies. Washington, D.C,2016; 20502. Reference Source

[14] DiCaprio E Phantkankum N Culbertson D: Inactivation of human norovirus and Tulane virus in simple media and fresh whole strawberries by ionizing radiation. *Int J Food Microbiol.* 2016;232:43–51. 10.1016/j.ijfoodmicro.2016.05.013 27240219

[15] World Institute for Nuclear Security: Considerations for the Adoption of Alternative Technologies to Replace Radioactive Sources.2016 Reference Source

[16] Industry Experts: Sterilization Technologies: A Global Market Overview.2016 Reference Source

[17] World Nuclear Association: The Many Uses of Nuclear Technology.2014; [cited 2016 October 28]. Reference Source

[18] Komolprasert V: Packaging for Foods Treated by Ionizing Radiation.2007 10.1002/9780470277720.ch6

[19] World Nuclear Association: Radioisotopes in Medicine.2016 Reference Source

[20] Interagency Working Group on Alternatives to High-Activity Radioactive Sources, Subcommittee on Nuclear Defense Research and Development, Committee on Homeland and National Security of the National Science and Technology Council: Transitioning from High-Activity Radioactive Sources to Non-Radioisotopic (Alternative) Technologies: A Best Practices Guide for Federal Agencies.2016 Reference Source

[21] Schmidt T Grabau D Grotewohl JH: Does sterilization with fractionated electron beam irradiation prevent ACL tendon allograft from tissue damage? *Knee Surg Sports Traumatol Arthrosc.* 2017;25(2):584–594. 10.1007/s00167-016-4240-9 27438006

[22] Rutala WA Weber DJ, Health care Infection Control Practices Advisory Committee (HICPAC): Guideline for Disinfection and Sterilization in Healthcare Facilities. Centers for Disease Control and Prevention.2008 Reference Source

[23] Gamma Industry Processing Alliance (GIPA): A Comparison of Gamma, E-beam, X-ray and Ethylene Oxide Technologies for the Sterilization of Medical Devices and Other Products.2017 Reference Source

[24] World Overview, Surgical Procedures (000s), in Pharmaceutical Fact Book.2012.

[25] Shekelle PG Wachter RM Pronovost PJ: Making health care safer II: an updated critical analysis of the evidence for patient safety practices. *Evid Rep Technol Assess (Full Rep).*Rockville MD.2013; (211):1–945. 24423049 PMC4781147

[26] US Food and Drug Administration: Guidance for Industry: Changes to an Approved NDA or ANDA. US Department of Health and Human Services: Washington, DC.2004 Reference Source

[27] Fölsch C Kellotat A Rickert M: Effect of thermodisinfection on mechanic parameters of cancellous bone. *Cell Tissue Bank.* 2016;17(3):427–37. 10.1007/s10561-016-9567-4 27344440

[28] Singh R Singh D: Sterilization of bone allografts by microwave and gamma radiation. *Int J Radiat Biol.* 2012;88(9):661–666. 10.3109/09553002.2012.700166 22671282

[29] Kubicek GJ Turtz A Xue J: Stereotactic Radiosurgery for Poor Performance Status Patients. *Int J Radiat Oncol Biol Phys.* 2016;95(3):956–959. 10.1016/j.ijrobp.2016.02.041 27113565

[30] Watanabe Y Lee R Dusenbery KE: Cumulative Dose to Brain After Multisession Gamma Knife Stereotactic Radiosurgery for Treatment of Multiple Metastatic Tumors. *Int J Radiat Oncol Biol Phys.* 2016;96(2):E83 10.1016/j.ijrobp.2016.06.801 PMC5859351 29594045

[31] Phan J Yang JN Ghia AJ: Dosimetric Comparison of Gamma Knife Extend to Linear Accelerator Base Volumetric Modulated Arc Therapy Plans for Fractionated Stereotactic Radiosurgery. *Int J Radiat Oncol Biol Phys.* 2015;93(3):E615–E616. 10.1016/j.ijrobp.2015.07.2118

[32] Lee WY Cho DY Lee HC: Outcomes and cost-effectiveness of gamma knife radiosurgery and whole brain radiotherapy for multiple metastatic brain tumors. *J Clin Neurosci.* 2009;16(5):630–4. 10.1016/j.jocn.2008.06.021 19269828

[33] Silverman E: FDA still struggling with backlog of generic drug applications. March 2, 2016, Dec 2, 2016. Reference Source

[34] Massdevice: Medical device makers spend millions to meet FDA rules, study finds. *Medcitizens* 2010 Reference Source

[35] International Atomic Energy Agency: Directory of Gamma Processing Facilities in Member States. Vienna, Austria,2004 Reference Source

[36] Bowerman BS Sullivan PT: Decommissioning the Brookhaven National Laboratory Building 830 Gamma Irradiation Facility.2001 10.2172/785049

[37] Pharmaceutical & Medical Packaging News: When in-house sterilization makes sense.1999 Reference Source

[38] International Atomic Energy Agency: National Regulatory Authorities with Competence in the Safety of Radiation Sources and the Security of Radioactive Materials. *International Conference of National Regulatory Authorities with Competence in the Safety of Radiation Sources and the Security of Radioactive Materials.*Buenos Aires, Argentina.2000 Reference Source

