# Supplementary material for: Unintended consequences of the potential phase-out of gamma irradiation
Source: F1000Res. 2018 Mar 21;7:348. [Version 1] doi: 10.12688/f1000research.14090.1 (PMC6058460; doi:10.12688/f1000research.14090.1)
Supplement: Peer review history

# Peer review: Referee response for version 1

**DOI:** 10.5256/f1000research.15326.r34941

**Published:** 2018-07-24

**Authors:** L. John Schreiner

**Affiliations:** 1 Departments of Oncology and Physics, Queen's University, Kingston, ON, Canada; 2 Cancer Centre of Southeastern Ontario at the Kingston Health Sciences Centre, Kingston, Ontario, Canada

The authors have presented quite a complete and compelling analysis of some unintentional and, perhaps, overlooked consequences for the USA and the world if use of the radioisotope cobalt-60 (Co‑60) is severely limited because of security motivated restrictions. They provide the motivation for controlling access to Co-60 sources in their review and analysis. And they lay out some consequences and risks through a good survey of the value of Co-60 in commerce, medicine and agriculture and the potential impact of restrictions. They reason that the restriction of Co-60 needs to be more carefully analysed, particularly in sterilization and medical applications, since other irradiation technologies and approaches are not yet fully developed for all these applications. They note that the ability to configure the radioactive Co-60 sources in purpose-built devices has provided robust solutions which may not be as easily, or economically, achieved with more technologically complex x-ray or electron irradiators.  And associated difficulties of increased complexity and decreased reliability may delay the transition to alternate approaches not yet in place. These are important points that have not been analysed broadly in the literature previously, and to bring them forward here presents a timely and important addition to the literature.

 Much of their discussion is limited to select applications in which Co-60 based irradiators have had a lead role in the United States and high income countries (HICs). And the analysis of these applications is quite good but there are two points I would suggest the authors consider in future work regarding the impact of the elimination of Co-60 treatment units in radiation therapy.

 1)   While the description in the paper on the implications of Gamma-knife restriction does give some context to the importance of Co-60 based brain cancer treatment, it has missed recent technological and clinical developments. The ability to treat small lesions and targets with high energy external beam radiation treatment (EBRT) has been extended to x-ray linear accelerator based units; this includes specialised devices and techniques such as small field robotic x-ray units (the CyberKnife, Accuray, Sunnyvale, CA) and advanced EBRT units with volumetric modulated arc radiotherapy (VMAT) capabilities that are now available in cancer clinics.  That is not to say that the Gamma-knife inventory currently deployed is not a critical resource. It is just to note that alternative devices are already available for this clinical application, although a transition to these devices would have considerable challenges in funding and clinical implementation.  

 2)    A further aspect of radioisotope restriction that could benefit from additional analysis is the impact of the removal of the Co-60 treatment units in low and middle income countries (LMICs) throughout the world. One can argue that the development of high photon energy Co-60 based devices (with effectively ~1.25 MeV mono-energetic beams) in the 1950s inaugurated high energy EBRT.  As noted by the authors, Co-60 radiation therapy devices have been essentially replaced in much of HICs (aside from select devices such as the Gamma-Knife, the recent ViewRay MRIdian Co-60 Co-60/MRI  unit, and some other specialised units). But over 2000 conventional Co-60 EBRT units are still in use in LMICs throughout the world, in large part because of the simple and robust design of these units and the low power and water supply infrastructure requirements for the devices (which often limit the feasibility of linear accelerator based x‑ray EBRT units in certain locales).  This is an important inventory already in place for cancer treatment, particularly in LMICs; the removal of these devices because of Co-60 security concerns may have critical negative effects.

 The World Health Organization has reported that LMICs annually account for over 60% of the world’s new cancer case, yet despite being home to 85% of the world’s population, they have less than 35% of the world’s radiotherapy facilities.   The increased  burden of cancer and the associated requirement for an increased distribution of EBRT radiation units in LMIC’S has also been highlighted by the International Atomic Energy Agency and the Global Task Force on Radiotherapy for Cancer Control. The restricted availability of Co‑60 devices potentially introduces a critical impediment to the important increased deployment of EBRT units as governments and international health agencies work to deal with this deficit. While research groups and radiotherapy vendors are working on developing simpler x-ray units for locales with limited infrastructure, it seems there will be a period over which we will rely on the simple Co-60 devices already deployed as well as additional installations (perhaps with new devices specially designed for improved performance and enhanced security of sources) to meet the cancer burden. And, as the authors discuss, the very countries requiring Co-60 radiotherapy deployment may be the same countries where Co-60 security is a challenge. Innovative solutions to ensure availability of needed Co-60 units with effective security control requires attention and the authors may be well set to undertake the analysis and discussion informing such solutions 

   

 I have also added some suggested references that discuss some points on Co-60 EBRT in LMICs.

I have read this submission. I believe that I have an appropriate level of expertise to confirm that it is of an acceptable scientific standard.

## References

[1] Van ScheltJSmithDLFongNToomehDSponsellerPABrownDWMacomberMWMayrNAPatelSShulmanASubrahmanyamGVGovindarajanKNFordEC: A ring-based compensator IMRT system optimized for low- and middle-income countries: Design and treatment planning study. *Med Phys*.2018;45(7) : 10.1002/mp.12985 3275-3286 10.1002/mp.12985 29777595PMC8522994

[2] DhanesarSDarkoJJoshiCPKerrASchreinerLJ: Cobalt-60 tomotherapy: clinical treatment planning and phantom dose delivery studies. *Med Phys*.2013;40(8) : 10.1118/1.4812417 081710 10.1118/1.4812417 23927307

[3] SchreinerLJJoshiCPDarkoJKerrASalomonsGDhanesarS: The role of Cobalt-60 in modern radiation therapy: Dose delivery and image guidance. *J Med Phys*.2009;34(3) : 10.4103/0971-6203.54846 133-6 10.4103/0971-6203.54846 20098559PMC2807677

# Peer review: Referee response for version 1

**DOI:** 10.5256/f1000research.15326.r32270

**Published:** 2018-04-17

**Authors:** Steven J. Goetsch

**Affiliations:** 1 San Diego Gamma Knife Center, San Diego, CA, USA; 2 Radiological Technologies University VT, South Bend, IN, USA

Jacqueline Chou and others have done an excellent job of evaluating the consequences to the United States from a ban of Co-60 gamma emitting radioisotopes in medicine and industry. The problem is actually far larger than that. Several more gamma emitting isotopes are also in widespread use. Gamma radiation from Co-60 is highly penetrating which makes it ideal for some applications and unsuitable for others. It is comparable in energy to a 4MV linear accelerator.

 Many medical devices contain Cesium-137. This isotope has a much longer half-life than Co-60 (30 years vs. 5.26 years) and emits gamma radiation with much lower energy, making it easier to shield. It is the isotope of choice for blood irradiators. Much of the US blood supply is irradiated before infusing into human patients, particularly those who are immune-compromised.

 Another extremely prevalent isotope is Ir-192.This isotope of iridium emits a multiplicity of gamma rays with about half the average energy of Cs-137 and one fourth the mean energy of Co-60 gamma radiation. Ir-192 is most commonly used in industrial radiography. There are literally thousands of such devices used in almost every major construction site in the country involving the use of structural steel. There is no other practical way to verify the competence of welding at a construction site which is vital to the safety of the bridges and buildings under construction.

 Another important field which makes extensive use of radioisotope sources is the oil and gas drilling industry.  Well logging sources (primarily Cs-137 and Americium-241 mixed with beryllium) are inserted into a well and reflected radiation is analyzed to determine the geologic structure of the layer being penetrated. 

 Each of these applications is important and very difficult to replace with radiation producing electronic devices.

I have read this submission. I believe that I have an appropriate level of expertise to confirm that it is of an acceptable scientific standard.
